# Hum Along With the Silent Disco Headphones: Lessons Learned in Implementing the Headphone Program in a Hospital Unit

**DOI:** 10.1093/geroni/igaa057.1217

**Published:** 2020-12-16

**Authors:** Lillian Hung

**Affiliations:** University of British Columbia, Vancouver, British Columbia, Canada

## Abstract

Silent disco headphones have been used among young people in concerts and parties; such headphones have extended distance coverage for broadcasting from a transmitter, features of noise cancelation, and three channels of music. Rather than using a speaker system, music is delivered by wireless headphones and facilitated by a DJ via a built-in microphone. No study has yet tested whether it is feasible to use such headphones to support well-being among older people in hospital settings. This study examined the feasibility of using silent disco headphones with older adults with dementia staying in a geriatric hospital unit. We employed a video-ethnographic design, including conversational interviews and observations, with video recording among ten patient participants in a hospital unit. Two focus groups were conducted with ten hospital staff across disciplines. Thematic analysis yielded three themes: (a) “it just made me feel happy, “(b) “it brings him back alive,” (c) “it unlocks dementia”. Delivering music and meditation programs via the silent disco headphones in the hospital unit has the potential to be a beneficial intervention that can enhance mood and energy, support self-expression, and promote wellness. Our findings suggested that witnessing the positive effects of headphones on patients changed the staff’s view of how music could be used in the clinical setting to support patients’ well-being. We identified enablers and barriers to implementing the headphone program in the hospital setting. Future research should further investigate how headphones may help to reduce stress and promote wellness for patients in the clinical environment.

